# Evaluation of Thoracoscopic Pericardial Window Size and Execution Time in Dogs: Comparison of Two Surgical Approaches

**DOI:** 10.3390/ani11051438

**Published:** 2021-05-18

**Authors:** Francesco Macrì, Vito Angileri, Claudia Giannetto, Lorenzo Scaletta, Piero Miele, Loris Pazzaglia, Simona Di Pietro

**Affiliations:** 1 Department of Veterinary Sciences, University of Messina, Viale Palatucci, 98168 Messina, Italy; Francesco.macri@unime.it (F.M.); vitoang@gmail.com (V.A.); claudiagiannetto@alice.it (C.G.); 2 Veterinaria Enterprise Stp S.R.L., Via Galvani 33d, 00153 Rome, Italy; lorenzo.scaletta@email.it (L.S.); mielepiero@gmail.com (P.M.); 3 Clinica Veterinaria Galilei, Via B. Franklin 22, 59100 Prato, Italy; info@clinicaveterinariagalilei.it

**Keywords:** canine, heart, pericardium, thoracoscopy

## Abstract

**Simple Summary:**

This canine prospective study was performed to statistically compare the surgery time and achieved windows size of two different thoracoscopic pericardiectomy techniques in dogs affected by pericardial effusion, using transdiaphragmatic paraxyphoid and monolateral intercostal approaches. The paraxifoid and the monolateral intercostal approaches showed a mean surgical time of 55 ± 20.08 (SD) minutes and 13.94 ± 4.61 (SD) minutes, and a mean pericardial window diameter of 4.23 ± 0.80 (SD) cm and 3.31 ± 0.43 (SD) cm, respectively. A significant correlation was observed between the dogs’ bodyweight and window size (r = 0.48; *p* = 0.04) for both surgical approaches, and between the dogs’ bodyweight and surgical time (r = 0.72; *p* = 0.0016) for monolateral intercostal approach. Our results provided useful information to help surgeons make the definitive choice of the surgical technique to treat the pericardial effusion.

**Abstract:**

Pericardial effusion presents clinicians with a challenge when diagnosing the underlying cause and performing a prognosis. Different techniques have been suggested for canine thoracoscopic pericardiectomy with the creation of variable pericardial window size. The aim of this study was to statistically compare the surgical time and achieved window size of the paraxiphoid transdiaphragmatic and monolateral intercostal approaches. The paraxifoid and monolateral intercostal approaches showed a mean surgical time of 55 ± 20.08 (SD) minutes and 13.94 ± 4.61 (SD) minutes, and a mean pericardial window diameter of 4.23 ± 0.80 (SD) cm and 3.31 ± 0.43 (SD) cm, respectively. A significant correlation was observed between the dogs’ bodyweight and window size (r = 0.48; *p* = 0.04) for both surgical approaches, and between the dogs’ bodyweight and surgical time (r = 0.72; *p* = 0.0016) for monolateral intercostal approach. All treated dogs showed no clinical signs of recurrent cardiac tamponade during the follow-up. Our results provided useful information to help surgeons make the definitive choice of the surgical technique to treat the pericardial effusion.

## 1. Introduction

Pericardial effusion is defined as an abnormal accumulation of fluid within the pericardial sac and can be a life-threatening emergency requiring rapid medical intervention. This condition presents clinicians with a challenge when diagnosing the underlying cause and performing a prognosis. In canine species, this condition has idiopathic, traumatic, septic or neoplastic origin [1,2,3,4].

In emergency medicine, pericardiocentesis is indicated for rapid relief of cardiac tamponade and patient stabilization. For long-term management of the pathology, surgical pericardiectomy is required so that the fluid can drain into the chest cavity reducing the intrapericardial pressure and cardiac compression [5,6,7].

Recently, minimally invasive procedures were used to perform the thoracoscopic pericardiectomy reducing the operating exposure, post-operative pain and convalescence, and providing long-term relief of clinical signs [8].

During the thoracoscopy, the visualization of intrathoracic structures and pathological lesions is superior to traditional open thoracotomy due to the excellent illumination and magnification of the image produced by the optics, allowing the examination of the previously inaccessible areas [9,10].

Different techniques have been suggested for canine thoracoscopic pericardiectomy, such as an approach with transdiaphragmatic paraxiphoid camera port and two operating ports on both left and right thoracic wall, or an approach with a single side port placement procedure [11,12,13,14,15,16].

The ideal size in centimeters for the pericardial window size has yet to be established [17], due to the variable heart size and different anatomic limits of various canine breeds.

The aim of this study was to statistically compare two different thoracoscopic pericardiectomies performed in 32 dogs affected by pericardial effusion, with a focus on the surgical time (minutes) and pericardial window size (cm). A paraxiphoid transdiaphragmatic approach (one paraxiphoid camera port and two instrument ports on the right and left side of the dog) and a monolateral intercostal right approach (two instrument ports on 4th and 8th intercostal spaces, and camera port on 6th space) were compared.

## 2. Materials and Methods

Thirty-two dogs affected by pericardial effusion were enrolled in this prospective study, conducted from January 2016 to January 2020. Informed consent was obtained from the owners.

Each dog, after hematological, hematochemical, radiographic, ultrasonographic examinations, and echo-hielded pericardial puncture, underwent thoracoscopic pericardiectomy.

Dogs were randomly divided into two groups. Group A was treated using a paraxiphoid transdiaphragmatic approach for optic trocar and two instrument ports placed in the 6th bilateral intercostal space. Group B was treated using a monolateral intercostal right approach with a camera port placement at the 6th intercostal space and the instrument ports at the 4th and 8th intercostal spaces.

The anesthetic protocol included the premedication with methadone 0.2 mg/kg IM, induction of anesthesia with propofol 4 mg/kg EV and maintenance with sevoflorane. Pain management was obtained performing an intercostal nerve block with bupivacaine or ropivacaine 0.25–0.5%. Intraoperative pain management with remifentanil or fentanyl was performed. During anesthesia, an anesthetic monitor recorded electrocardiography, heart and respiratory rate, end-tidal CO_2_, saturation of blood O_2_ and non-invasive blood pressure.

The same surgeon with a high level of experience in thoracic surgery and minimally invasive surgical techniques performed all endoscopic procedures.

In both groups, operative instruments included a Tele Pack Vet X Led system, 5 mm 30° telescope (Karl Storz Endoscopia Italia S.r.l., Verona, Italy), 6 mm cannulae (Karl Storz Endoscopia Italia S.r.l., Verona, Italy), curved scissors (Karl Storz Endoscopia Italia S.r.l., Verona, Italy) attached to bipolar electrocoagulation, and grasping forceps (Karl Storz Endoscopia Italia S.r.l., Verona, Italy).

In Group A, each dog was positioned in dorsal recumbency and hair was clipped bilaterally from the second intercostal space to the hypochondrium region. The site was then prepared and draped for aseptic surgery. Thoracoscopic pericardial window was performed in all dogs using a three-cannula technique. A 6 mm cannula was placed in right paraxiphoid position with an inclination of 45° in cranio-lateral direction. A 5 mm 30° telescope was placed through this cannula to view the thoracic cavity. A pneumothorax was established and then a 6 mm cannula was placed for instrument placement at the left sixth intercostal space ventrally to the costochondral junction. Curved scissors attached to bipolar electrocoagulation was used to create a hole in the ventral mediastinum through which it was possible to visualize the right hemithorax and a 6 mm instrument cannula was positioned at the right sixth intercostal space, ventral to the costochondral junction. The sternopericardial ligament was then sectioned in order to obtain the widest possible view of the apex of the heart. After visualization of the cranio-ventral surface of the pericardium, it was grasped using grasping forceps and curved scissors were used to create an initial hole on the pericardium, as dorsal as possible. Then a pericardial window as wide as possible was obtained. The excised pericardium was extracted from the thoracic cavity and measured. At the end of the surgical procedure, to evacuate the pneumothorax postoperatively, a thoracic drain was placed.

In Group B, each dog was positioned in dorsal recumbency and then was clipped, scrubbed and draped on the right side of the chest, from midway sternum to the dorsal portion of the thoracic cage. Operative instruments were inserted through a 6 mm cannula placed in the 6th intercostal space with an inclination of 30°. A 5 mm 30° telescope was placed through this cannula to view the thoracic cavity. After the insertion of the trocar and the consequent formation of the open pneumothorax, thoracotomy for the instrument ports placement was performed at the 4th and 8th intercostal spaces in lower position than camera. Pericardial window was performed with forceps and scissors obtaining a piece of the pericardium as wide as possible that was extracted from the thoracic cavity and measured. A thoracic drain was placed at the end of the surgical procedure.

In all dogs, an excessive pericardial thickening or adherences that could influence the length of surgery were not observed.

For each group of enrolled dogs, the execution time of surgery (minutes) and the achieved size of the pericardial window (diameter, centimeters) were evaluated. Surgery time was defined as total time spent from the initial port placement to closure of pericardiectomy procedure.

After the surgery, a follow-up at 7, 30, 90, and 150 days was performed for all dogs. During the examination, the ultrasound assessment of a cardiac tamponade recurrence was performed.

### Statistical Analysis

Data were normality distributed (*p* > 0.05; Pearson normality test). Unpaired Student t-test was applied to determine whether significant differences existed between the bodyweight of dogs treated by the pericardiectomy with a paraxifoid or monolateral intercostal approach, and to evaluate differences in the surgical time and window size between the groups. The relationship between the window size and surgical time was evaluated using linear regression analysis. A correlation coefficient (Pearson r) was evaluated for each surgical approach. The relationship between dogs’ bodyweight and the investigated variables (surgical time and window size) was also evaluated through the correlation test. *p* values < 0.05 were considered statistically significant. Data were analyzed using statistical software Prism v.5.00 (GraphPad Software Ltd., San Diego, CA, USA, 2003).

## 3. Results

Group A included 16 dogs with ages ranging from 5 to 13 years. Mean body weight was 23.88 ± 11.46 (SD) Kg (range 4–45). The breeds were Beagle (1), Mongrel dog (3), Pomeranian (1), Pug (1), Hound (1), German Shepherd (3), Boxer (2), Corso (1), English Setter (1) and Golden Retriever (2). Group B included 16 dogs with age ranged from 6 to 12 years, of different breeds: Boxer (4), Bernese Mountain Dog (1), Beagle (1), Great Dane (1), Schnauzer (1), Labrador Retriever (2), Mastiff (1), Cocker Spaniel (1), Bolognese (1), Rottweiler (1), German Shepherd (1) and Mongrel dog (1). The mean body weight was 30 ± 13.61 (SD) Kg (range 4–58).

Unpaired Student t-test showed no statistical differences in dogs’ bodyweight between the two groups (*p* = 0.17).

The most common clinical signs in enrolled dogs were lethargy, difficult breathing, anorexia or decreased appetite and cough. Physical examination revealed muffled heart sounds, weak femoral pulses and tachypnea. No consistent abnormalities were found on the complete blood count or serum biochemical profile. An enlarged cardiac silhouette was observed in all dogs performing thoracic radiographs. Ultrasonographic evaluation of the heart confirmed pericardial effusion in all cases.

Table 1 shows the statistic column analysis for the investigated variables (surgery time and window size) in each experimental surgical approach.

**Figure 1:**
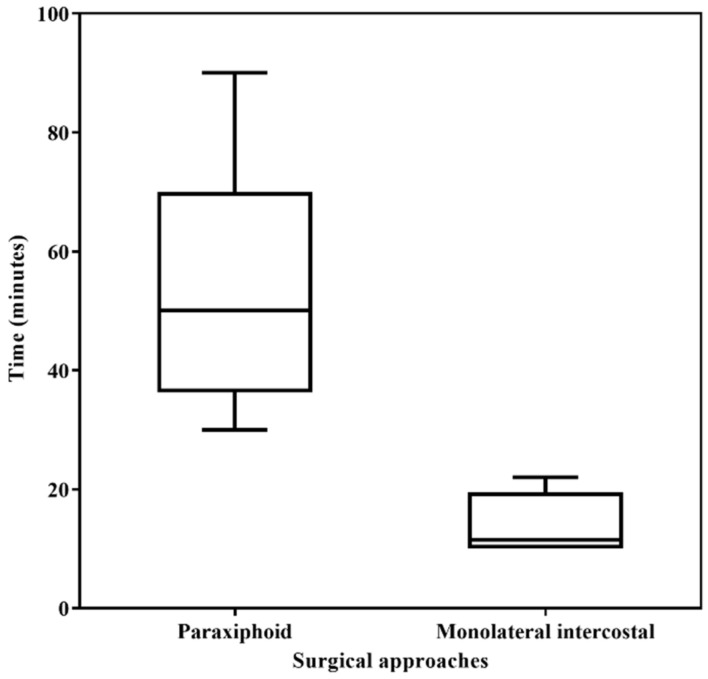
Comparison of the surgery time between two thoracoscopic pericardial techniques (paraxifoid approach vs. monolateral intercostal approach). Box-plot depicts time’s minimum and maximum (whiskers) and medians (line across the box).

The paraxiphoid transdiaphragmatic approach was significantly longer (mean surgery time 55 ± 20.08 (SD) minutes) than the monolateral intercostal approach (mean 13.94 ± 4.61 (SD) minutes) (*p* < 0.0001) (Figure 1).

**Figure 2:**
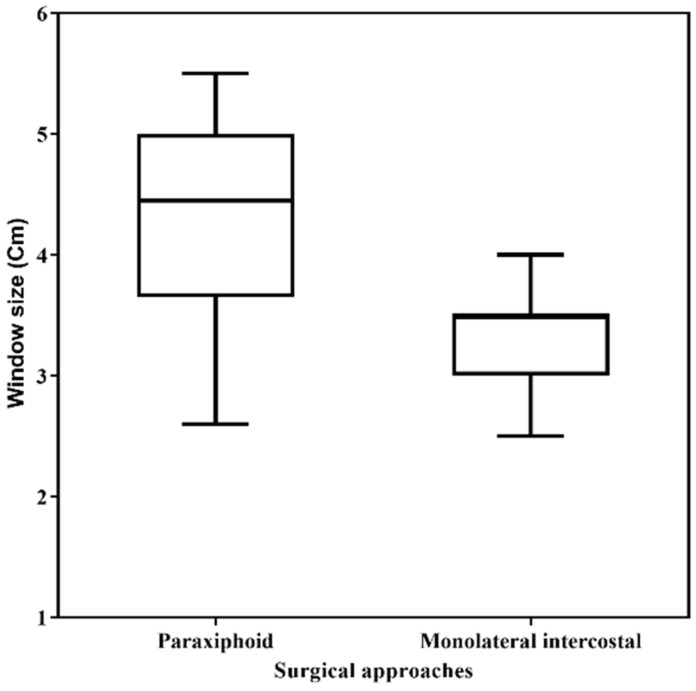
Comparison of the pericardial window size between two thoracoscopic pericardial techniques (paraxifoid technique vs. monolateral intercostal technique). Box-plot depicts window size’s minimum and maximum (whiskers) and medians (line across the box).

The paraxiphoid approach resulted in a significantly larger window size (mean diameter 4.23 ± 0.80 (SD) cm) compared to monolateral intercostal technique (mean diameter 3.31 ± 0.43 (SD) cm) (*p* < 0.0001) (Figure 2).

**Figure 3:**
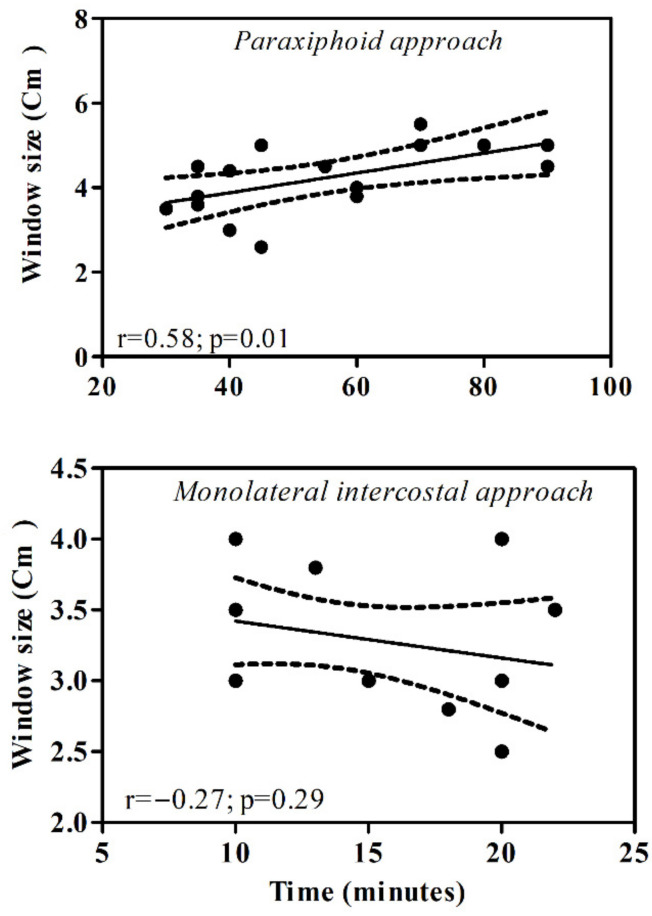
Scatter plot of the surgery time vs. pericardial window size in each surgical treatment. The distribution data showed a linear regression for the paraxiphoid approach.

In the study population, there was a linear correlation between the time spent for the paraxiphoid procedure and the dimension of the pericardial window (r = 0.58; *p* = 0.01). Surgery time increased with increasing the size of the created window (Figure 3). In Group A, a significant correlation was also observed comparing the dogs’ bodyweight versus the surgical time (r = 0.72; *p* = 0.0016) and versus the window size (r = 0.48; *p* = 0.04). In Group B, a significant correlation was observed between the dogs’ bodyweight and the window size (r = 0.74; *p* = 0.001), but not between the dogs’ bodyweight and surgical time (r = 0.06; *p* = 0.82).

All treated dogs showed no clinical signs of recurrent cardiac tamponade during the follow-up examination until 150 days after the surgery.

## 4. Discussion

Pericardial effusion, irrespective of the etiology, can result in cardiac tamponade so that to find an efficient method of drainage is mandatory.

Thoracoscopic pericardiectomy with the creation of a pericardial window is considered as a definitive treatment for idiopathic pericardial effusion or palliative therapy for malignant lesions [8,11].

This study allowed us to assess two critical points of the management of canine pericardial effusion as the surgery time and the achieved pericardial window size, comparing two thoracoscopic pericardiectomy procedures frequently used in our clinical practice.

The study groups reflect the type and variety of cases presented to veterinary surgeons where clinical and therapeutic decisions must be made.

Various factors can complicate the decision-making process about the more suitable surgical approach, such as the experience and training of the surgeon or surgical team, the intraoperative equipment availability, as well as the postoperative support [14,17].

The objective of this study was to provide reliable data, based on the authors’ experience, to help the surgeon choose the surgical approach for each patient affected by pericardial effusion.

The statistical analysis performed in this study showed no difference between dogs’ bodyweight in the two groups. Moreover, a direct correlation between the surgery time and window size with the dogs’ bodyweight for the paraxifoid techinque was observed, while the time spent to perform the monolateral intercostal technique was independent by the patients’ size. In the latter technique, when the surgical time increases, the size of the pericardiectomy is smaller. This finding could be associated with a more complex but not less effective procedure in small-sized breed dogs. In this study, since no recurrence of cardiac tamponade was found during the follow-up, both procedures were found to be effective.

Recommendations regarding ideal pericardial window diameter are limited, and this varies by dog size, as demonstrated by the statistical correlation in both groups. A significantly longer surgical time has been needed in the paraxifoid approach to obtain a wider window (4.23 cm on average) when compared to the lateral approach. This difference has been not clinically relevant, as demonstrated during the follow-up.

The longer surgical time of the paraxifoid approach could be related to the technical feasibility. In large breeds, working from both flanks can be uncomfortable, hampering the maneuverability and affecting the surgery length.

The paraxiphoid approach might be recommended for idiopathic effusion because it offers a wider vision of the thoracic cavity and eases the exploration searching for possible sources of the effusion like foreign bodies.

On the other hand, the monolateral intercostal approach could more easily perform a pericardial fenestration, providing a shorter time of execution independent of the dog size. One of the reasons to attempt a monolateral approach could be to avoid having to reposition the patient after performing another procedure (i.e., thoracic duct surgery) in sternal or lateral recumbency. Further studies could be interesting to compare the monolateral intercostal approach with the patient in sternal or lateral recumbency. According to the literature [9,11], both thoracoscopic pericardiectomy approaches were performed maintaining the dog in dorsal recumbency. This choice allowed the best visualization and access to thoracic structures, avoiding selective intubation and maintaining bilateral lung function.

The current study presents some limitation. The area of the surgically created pericardial window may have been larger than the measured area of the resected pericardium, due to its contraction to a smaller size following extraction from the thoracic cavity. Unfortunately, no images were taken to measure the diameter of the window created in situ.

Another limitation of this study is the impossibility to blind the surgeon as to which approach is used. Thus, potential bias in both the timing and size of the window is possible.

In this study, the relationship between the etiology and the surgical time within the same group was not performed. Futures studies on this topic would be very interesting.

## 5. Conclusions

This study allowed us to evaluate the difference between the paraxiphoid and monolateral intercostal pericardiectomies for the management of dogs affected by pericardial effusion, with a focus on the time spent for the surgical procedure and the achieved windows size.

Our results provided useful information to help surgeons make the definitive choice of the surgical technique to treat the pericardial effusion.

## Figures and Tables

**Table 1:** Statistic column analysis for the investigated variables (surgery time and window size) in each experimental surgical approach (paraxiphoid and monolateral intercostal).

Approach	Paraxiphoid		Monolateral Intercostal	
Experimental Variable	Surgical Time	Window Size	Surgical Time	Window Size
Minimum	30.000	2.60	10.00	2.50
25% Percentile	36.25	3.65	10.00	3.00
Median	50.00	4.45	11.50	3.50
75% Percentile	70.00	5.00	19.50	3.50
Maximum	90.00	5.50	22.00	4.00
Mean	55.00	4.23	13.94	3.31
Std. Deviation	20.08	0.80	4.61	0.43
Std. Error	5.02	0.20	1.15	0.10
Lower 95% CI of mean	44.30	3.80	11.48	3.08
Upper 95% CI of mean	65.70	4.66	16.39	3.54

## Data Availability

The data presented in this study are available on request from the corresponding author.
